# Quantitative affinity purification mass spectrometry: a versatile technology to study protein–protein interactions

**DOI:** 10.3389/fgene.2015.00237

**Published:** 2015-07-14

**Authors:** Katrina Meyer, Matthias Selbach

**Affiliations:** Proteome Dynamics, Max Delbrück Center for Molecular Medicine, Berlin, Germany

**Keywords:** mass spectrometry based proteomics, quantitative proteomics, protein–protein interaction, stoichiometry, cross-linking

## Abstract

While the genomic revolution has dramatically accelerated the discovery of disease-associated genes, the functional characterization of the corresponding proteins lags behind. Most proteins fulfill their tasks in complexes with other proteins, and analysis of protein–protein interactions (PPIs) can therefore provide insights into protein function. Several methods can be used to generate large-scale protein interaction networks. However, most of these approaches are not quantitative and therefore cannot reveal how perturbations affect the network. Here, we illustrate how a clever combination of quantitative mass spectrometry with different biochemical methods provides a rich toolkit to study different aspects of PPIs including topology, subunit stoichiometry, and dynamic behavior.

## Introduction

Proteins do not act in isolation but typically mediate their biological functions by interacting with other proteins (Charbonnier et al., 2008). Owing to the central importance of protein–protein interactions (PPIs) in biology, methods have been developed to study multiple aspects of PPIs (Meyerkord and Fu, 2015). For example, X-ray crystallography and NMR provide detailed spatial information about interaction interfaces. Surface plasmon resonance (SPR), isothermal titration calorimetry (ITC), and förster resonance energy transfer (FRET) provide binding affinities and kinetics. However, all of those methods require *a priori* knowledge of the interaction partners and suffer from the drawback of a low throughput. Technologies like protein microarrays, phage display and the yeast two-hybrid system permit high-throughput screens for PPIs. However, these approaches rely on *in vitro* assays or heterologous biological systems. Therefore, it is not clear if PPIs detected by these methods occur in the relevant *in vivo* context.

Affinity purification combined with mass spectrometry (AP-MS) has emerged as a particularly attractive method for PPI mapping (Gingras et al., 2007). A major advantage is that this method allows unbiased detection of PPIs under physiological conditions. Importantly, AP-MS can assess PPIs in relevant biological contexts such as mammalian cell lines or even tissues. Moreover, AP-MS experiments have the advantage that they can provide quantitative information (q-AP-MS). This greatly increases the confidence in interaction partners that are identified and can also be used to study the impact of perturbations on PPIs.

We argue that q-AP-MS is one of the most powerful technologies to map PPIs in health and disease. The aim of this Mini Review is to briefly explain the general principle of q-AP-MS and to emphasize the versatility of AP-MS to investigate various aspects of PPIs including quantities, topology, subunit stoichiometry, and dynamic behavior. We will begin with a brief introduction to quantitative shotgun proteomics.

## Quantitative Shotgun Proteomics

The principle idea of shotgun proteomics is that protein samples are first digested into peptides (Aebersold and Mann, 2003). These peptides are then separated by high performance liquid chromatography (HPLC) and directly (“online”) transferred into a mass spectrometer. This instrument performs two important tasks. First, it measures the mass to charge ratios (m/z) and intensity of the peptides eluting from the HPLC column (MS^1^). Second, in order to determine the amino acid sequence, the instruments selects individual peptides for fragmentation and records the resulting fragment spectra (MS^2^). Data generated in this manner is then compared to protein databases for peptide and protein identification (Eng et al., 2011).

**FIGURE 1:**
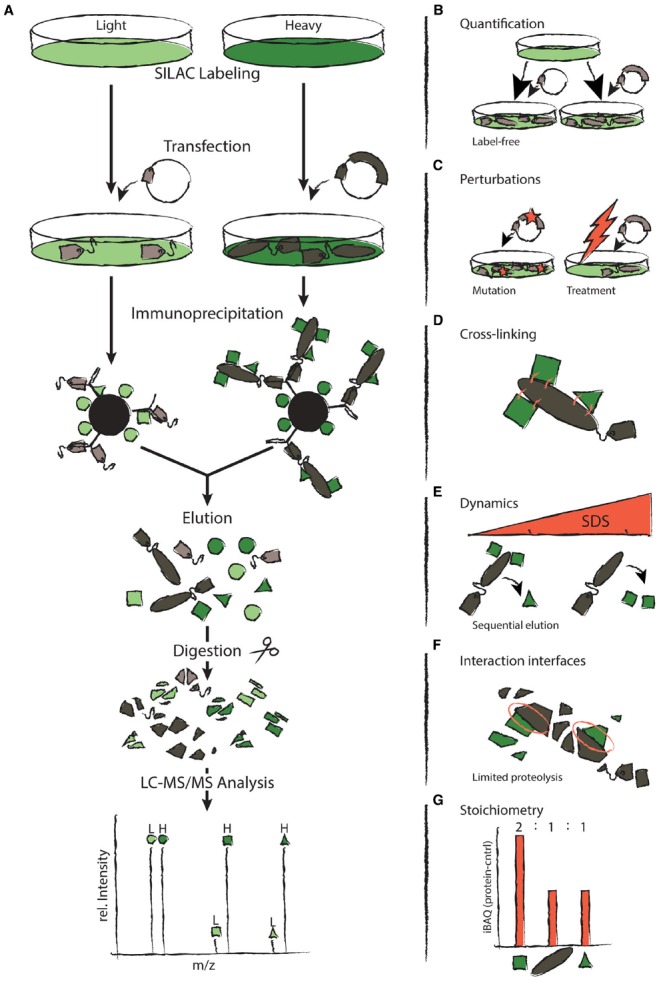
**Approaches to q-AP-MS experiments. (A)** The left hand side depicts the typical workflow of a SILAC-based q-AP-MS experiment. Differentially SILAC-labeled cells are transfected with a tagged protein of interest or a control vector containing only the tag, respectively. Proteins are immunoprecipitated with antibodies directed against the tag. Samples are mixed prior to elution. Eluted proteins are cleaved into peptides and analyzed by Liquid-Chromatography Mass Spectrometry (LC-MS). **(B–G)** The right hand side depicts how q-AP-MS can be employed to study different aspects of PPIs. **(B)** Label-free quantification provides an alternative to SILAC. **(C)** Immunoprecipitation can compare changes in PPIs upon perturbation. **(D)** Transient interactions and complex structure can be studied by cross-linking. **(E)** Submodule composition and PPI dynamics can be revealed by sequential elution with increasing concentrations of SDS. **(F)** Limited proteolysis provides a means to detect interaction interfaces. **(G)** The stoichiometry of complexes can be revealed by comparing abundances of the different subunits.

Until a decade ago, the field of proteomics has used mass spectrometry mainly to draw qualitative conclusions about the existence of a protein in a given sample. The reason for this is that the intensities of peaks in a mass spectrum are not directly proportional to the amounts of the corresponding peptides. Hence, mass spectrometry is intrinsically not a quantitative technology (Ong and Mann, 2005). However, over the past several years various technologies have been developed to enable proteome-wide quantification using mass spectrometry (Gstaiger and Aebersold, 2009; Cox and Mann, 2011; Bantscheff et al., 2012). One idea relies on the incorporation of stable heavy isotopes into proteins through metabolic (SILAC) or chemical labeling approaches. This permits different cell populations to be mixed and analyzed together, since the mass-shift introduced by the labeling makes them distinguishable. Relative changes in peptide intensities reflect differences in the abundance of the proteins under distinct experimental conditions. Alternatively, proteins can be quantified using computational methods (“label-free quantification”; Figure 1B). This may be based solely on how often peptides have been chosen for fragmentation (spectral counting) or on all intensities obtained from precursor peptide scans. Care should be taken when employing the first approach, since it provides only very rough abundance estimates (Rinner et al., 2007; Gingras and Raught, 2012). While the choice of a quantification approach depends on various factors, stable isotope-based methods are generally more precise than label free approaches since samples can be combined and analyzed together (Sury et al., 2010; Lau et al., 2014). For example, while stable isotope-based methods can detect even minor changes in protein abundance, label free approaches typically require a twofold change or more (Cox et al., 2014).

## Specificity and Sensitivity

A major challenge in AP-MS is to distinguish true interaction partners from non-specific contaminants. An early idea to address this problem was tandem affinity purification (TAP; Puig et al., 2001). Here, the protein of interest is expressed as a fusion with two different biochemical tags. Two consecutive rounds of affinity purification are then employed in order to remove non-specific contaminants. Although this approach has been used successfully in many studies, it has two major disadvantages. First, only very stable complexes survive the procedure, which means that TAP cannot be used to study more dynamic interactions. Second, the sensitivity of modern mass spectrometers is so high that they still detect many non-specific binders after TAP. An alternative idea is to use a single purification step and to exclude non-specific contaminants based on prior knowledge. The “contaminant repository for affinity purification” (CRAPome) was built for this purpose and contains information about frequently observed unspecific binders (Mellacheruvu et al., 2013). While this is generally a good idea, one important limitation is that the non-specific background depends on specific experimental conditions. In other words, not all proteins in the CRAPome are necessarily contaminants in a specific experiment, nor are all contaminants in a specific experiment contained in the CRAPome.

Quantitative proteomics offers an attractive solution to address these challenges (Figure 1A). In quantitative AP-MS (q-AP-MS), the quantity of proteins that co-purify with the bait is compared to a negative control (Vermeulen et al., 2008; Paul et al., 2011). In this set-up, true interaction partners can be identified by their specific abundance ratio while non-specific contaminants bind equally well under both conditions, which results in a 1:1 ratio. Hence, q-AP-MS uses quantification to filter out non-specific contaminants. This greatly increases confidence in identified interaction partners, even under mild biochemical purification conditions.

## Perturbations

One of the major advantages of q-AP-MS is that it can assess dynamic changes in PPIs upon perturbation (Figure 1C). To this end, the proteins which co-purify with a bait protein under normal and perturbed conditions are compared in a quantitative manner. An early example of this general principle employed the immobilized SH2-domain of the adapter protein Grb2 to study epidermal growth factor (EGF) receptor signaling (Blagoev et al., 2003). SH2 domains interact with specific tyrosine-phosphorylated motifs. Therefore, the immobilized domain was used in cells stimulated with EGF to pull down interacting proteins. Cells that had not been stimulated served as a negative control. Subsequently, a quantitative comparison of the two pull-down contexts revealed proteins recruited to Grb2 upon activation by EGF. After this pioneering work, the same idea was used to assess dynamic PPIs during cell signaling with different experimental designs. For example, immobilized peptides carrying specific posttranslational modifications and their unmodified counterparts were used to identify modification-dependent interactions (Selbach et al., 2009; Bartke et al., 2010; Francavilla et al., 2013). Immunoprecipitation of endogenous or epitope-tagged proteins before and after stimulation has also been frequently employed (Collins et al., 2013; Zheng et al., 2013; Sury et al., 2015). Finally, quantification can reveal differences in the interaction partners of wild-type proteins and disease-associated variants (Lambert et al., 2013; Hosp et al., 2015). If those mutations map to a protein with unknown function, an AP-MS experiment can provide valuable insights based on the known functions of identified interaction partners.

## *In vivo* Interactions

Many AP-MS studies make use of overexpressed and/or tagged proteins as baits. However, this may interfere with the normal *in vivo* function of the protein and thus lead to false-positive or false-negative results. Overexpression artifacts can be limited when tagged proteins are expressed at near-endogenous levels, for example using bacterial artificial chromosomes (Hubner et al., 2010). However, it is still possible that the tag interferes with protein function. It has been shown recently that even cloning scars between the protein and the tag can lead to false-positive identifications (Banks et al., 2015). This problem can be addressed by targeting the endogenous protein with specific antibodies. While this has been employed successfully (Malovannaya et al., 2011; Lundby et al., 2014), an important caveat is that antibody cross-reactivity may lead to false-positive results. In case of tagged proteins the specificity can be assessed using untransfected cells as negative controls, but this is not possible when the endogenous protein is targeted. To address this issue, many published studies have used control antibodies. However, due to differences in the cross-reactivity of various antibodies, this strategy is questionable. A better control is to knock down the protein of interest in the control condition, which makes it possible to use the same antibody for comparison (Selbach and Mann, 2006). Nevertheless, the lack of good antibodies is an important limitation and one of the reasons why epitope-tagged proteins still dominate such studies.

Another important consideration is that the interaction partners identified in cell lines may not necessarily be relevant *in vivo*. More and more studies therefore purify proteins and their interaction partners directly from animal models (Cheeseman et al., 2004; Angrand et al., 2006; Bartoi et al., 2010; Rees et al., 2011; Hanack et al., 2015). With the advent of genome editing techniques such as CRISPR it is now possible to generate genomic tag knock-ins in an efficient manner (Sander and Joung, 2014). This makes it much easier to create tagged versions of endogenous proteins for *in vivo* interactome mapping and tissue culture experiments. Most of the methods discussed here are generally applicable to any organism. Even the SILAC approach, which was originally developed for metabolic labeling of tissue culture cells, has since been extended to a number of model organisms (Kirchner and Selbach, 2012). Thus, we expect that *in vivo* interaction proteomics will become more widespread.

## Cross-linking

Upon cell lysis, proteins are brought into an artificial environment. This can result in the loss of weak or transient interactions or the formation of *in vitro* interactions in the lysate. One way to address this problem is *in vivo* cross-linking (Kaake et al., 2014; Figure 1D). Newly formed covalent bonds between interacting proteins permit stringent purification conditions which minimize *in vitro* interactions and preserve transient interactions (Tardiff et al., 2007; Fang et al., 2012). Moreover, the identification of cross-linked peptides can provide valuable information about the structure of proteins and complexes (Rappsilber, 2011; Walzthoeni et al., 2013). Despite these advantages, most AP-MS experiments performed today do not employ cross-linking. One reason is that cross-linked peptides are typically less abundant and are thus more difficult to identify than regular peptides. To address this problem, several strategies that enrich for cross-linked peptides have been developed (Rinner et al., 2008; Nessen et al., 2009).

## Interaction Interfaces

Cross-linking requires that target sites be accessible, which makes it difficult to apply this approach to interfaces buried within a protein complex. This limitation is actually used as an advantage in several other methods to provide information about interaction interfaces. For example, protein painting employs small molecular dyes which adhere to the accessible surfaces of protein complexes, excluding binding interfaces (Luchini et al., 2014). During the subsequent digestion, only peptides within interaction interfaces are accessible to trypsin and can thus be identified. Limited proteolysis (Feng et al., 2014) is an approach that is complementary to protein painting, in that it reveals only peptides outside interaction interfaces that are accessible to trypsin (Figure 1F). Another possibility is to treat samples with heavy (i.e., deuterated) water: hydrogen-deuterium exchange (HDX; Mandell et al., 2005) relies on the fact that amides hidden within protein–protein interfaces are not in direct contact with the solvent and will exchange their hydrogen atoms at a lower rate than more accessible amides. The corresponding changes in the peptide mass can then be detected using mass spectrometry. These techniques are not only useful in the study of PPIs but can additionally provide information about protein structure (Chorev et al., 2015).

## Stoichiometry

The approaches mentioned above typically rely on relative quantification. Thus, they can be used to distinguish specific interaction partners from contaminants and to quantify dynamic changes in PPIs upon perturbation. However, these methods can only compare the same protein under different conditions. They do not provide information about the stoichiometry of the distinct members of a complex. One way to compare different proteins in a complex is to measure their absolute abundances using synthetic isotope-labeled reference peptides as spike-in standards (Schmidt et al., 2010). For a large number of proteins, this is tedious and expensive. The SH-quant approach therefore incorporates an additional reference peptide into the affinity tag that is used for the pull-down (Wepf et al., 2009). This permits quantification of the bait and also of prey proteins, in the event they have been used as baits in another experiment. This “correlational quantification” allows the measurement of protein complex stoichiometry and absolute protein complex abundances. Alternatively, the stoichiometry of protein complexes can also be analyzed through a combination of affinity purification and intensity-based absolute quantification (iBAQ; Figure 1G; Schwanhausser et al., 2011; Smits et al., 2013). The latter approach has the advantage that it is easy to implement and does not require the tagging of multiple baits. It is also important to keep in mind that the same bait protein can be part of multiple protein complexes. Therefore, not all proteins that co-purify with a bait are necessarily members of the same complex. Distinguishing between these different complexes requires the individual pull-down of all components.

## Dynamic Interactions

Not all of the specific interaction partners of a protein necessarily belong to a stable complex. Some interaction partners interact only transiently. The dynamic behavior of proteins can be investigated by mixing protein samples at different stages of an AP-MS experiment. Metabolic labeling approaches such as SILAC allow a mixing of samples directly after cells are harvested (Ong et al., 2002). While this minimizes experimental differences in sample handling, it also results in the loss of dynamic interactions with high on/off rates: During incubation with antibodies, these dynamic interaction partners will be exchanged between both conditions and reach equilibrium over time. Alternatively, samples may first be mixed after affinity purification. When both protocols are performed in parallel on the same samples, the data can be used to identify the dynamic components in protein complexes (Mousson et al., 2008; Wang and Huang, 2008). A related idea uses increasing concentrations of SDS to elute precipitated proteins sequentially (Figure 1E; Texier et al., 2014). These data can be used to dissect the submodular composition of complexes due to their different binding properties.

Binding affinity is a particularly relevant quantity with regard to characterizing the interaction between two proteins. Typically, binding affinities are measured using methods such as ITC or SPR assays which require considerable quantities of purified proteins. q-AP-MS experiments can also be designed in a way to provide information about binding affinities (Sharma et al., 2009): First, a known quantity of an immobilized bait is incubated with cell extracts to pull down interactors. Next, the supernatant from this experiment is used in a second pull-down with the same bait. The quantification of the proteins in both pull-downs can then be used to infer the dissociation constants of the interactions. While so far this technique has only been used to calculate equilibrium dissociation constants (K_d_s) of proteins interacting with small molecules and peptides, it should be generally applicable to a range of ligands, including entire proteins, used as baits.

## Conclusions

The examples described above show that a combination of quantitative shotgun proteomics with various biochemical methods can provide a rich toolkit to explore various aspects of PPIs. This can be employed to (i) identify binding partners with high specificity, (ii) assess the stoichiometry of complexes, (iii) provide information about interaction interfaces, (iv) analyze binding affinities, and (v) study dynamic changes of PPIs upon perturbation. Bearing in mind possible pitfalls (Duncan et al., 2010), mass spectrometers can thus be regarded as “Swiss army knives” for PPI research. Since instruments are becoming faster, more sensitive, easier to operate and cheaper, we expect these approaches to become available to more and more scientists.

### Conflict of Interest Statement

The authors declare that the research was conducted in the absence of any commercial or financial relationships that could be construed as a potential conflict of interest.
